# Coronavirus disease 2019: A tissue engineering and regenerative medicine perspective

**DOI:** 10.1002/sctm.20-0197

**Published:** 2020-08-21

**Authors:** Abbas Shafiee, Lida Moradi, Mayasari Lim, Jason Brown

**Affiliations:** ^1^ Herston Biofabrication Institute Metro North Hospital and Health Service Brisbane Queensland Australia; ^2^ Royal Brisbane and Women's Hospital Metro North Hospital and Health Service Brisbane Queensland Australia; ^3^ UQ Diamantina Institute, Translational Research Institute The University of Queensland Brisbane Queensland Australia; ^4^ Department of Cell Biology New York University, School of Medicine New York New York USA; ^5^ The Ronald O. Perelman Department of Dermatology New York University, School of Medicine New York New York USA; ^6^ RoosterBio Frederick Maryland USA

**Keywords:** acute respiratory distress syndrome, biomaterial, cell processing, clinical trials, mesenchymal stem cell

## Abstract

Current therapies for novel coronavirus disease (COVID‐19) are generally used to manage rather than cure this highly infective disease. Therefore, there is a significant unmet medical need for a safe and effective treatment for COVID‐19. Inflammation is the driving force behind coronavirus infections, and the majority of deaths caused by COVID‐19 are the result of acute respiratory distress syndrome (ARDS). It is crucial to control the inflammation as early as possible. To date, numerous studies have been conducted to evaluate the safety and efficacy of tissue engineering and regenerative medicine (TERM) products, including mesenchymal stem cells (MSCs), and their derivatives (eg, exosomes) for coronavirus infections, which could be applied for the COVID‐19. In this review, first, the impacts of the COVID‐19 pandemic in the present and future of TERM research and products are briefly presented. Then, the recent clinical trials and the therapeutic benefits of MSCs in coronavirus‐induced ARDS are critically reviewed. Last, recent advances in the field of tissue engineering relevant to coronavirus infections, including three‐dimensional platforms to study the disease progression and test the effects of antiviral agents, are described. Moreover, the application of biomaterials for vaccine technology and drug delivery are highlighted. Despite promising results in the preclinical and clinical applications of MSC therapy for coronavirus infections, controversy still exists, and thus further investigation is required to understand the efficacy of these therapies.

## Graphical abstract

The recent clinical trials and the therapeutic benefits of mesenchymal stem cells in coronavirus‐induced acute respiratory distress syndrome are critically reviewed. Then, the new advances in the field of tissue engineering relevant to the coronavirus infections, including three‐dimensional models, to study disease progression or test the antiviral agents are described. Moreover, the potential applications of biomaterials for vaccine technology, and drug delivery are highlighted.

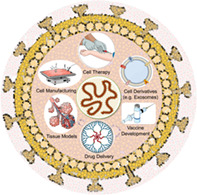

**Significance statement**

The tissue engineering and regenerative medicine communities and industries have been largely impacted by the COVID‐19 pandemic. In this article, the impact of the recent pandemic on the present and future of tissue engineering and regenerative medicine research and therapies is highlighted. Then, the potential use of three‐dimensional tissue models and benefits and risks of mesenchymal stem cell therapy for the COVID‐19 are discussed.

## 1 INTRODUCTION

The outbreak of a novel coronavirus (2019‐nCoV) in late December 2019 has led to a global pandemic known as novel coronavirus disease (COVID‐19). As of 10 June 2020, over 7 400 000 confirmed cases and over 410 000 deaths have been reported worldwide, and numerous businesses are being impacted by COVID‐19. The healthcare communities and industries have been largely impacted by this pandemic. In this article, the impact of the recent pandemic on the present and future of tissue engineering and regenerative medicine (TERM) research and therapies is highlighted. Then, the potential use of three‐dimensional (3D) tissue models and benefits and risks of cell therapy approaches, stem cells specifically, for the COVID‐19 are discussed.

## 2 IMPACT OF COVID‐19 PANDEMIC ON TERM RESEARCH AND THERAPIES

### 2.1 Majority of non‐COVID‐19 clinical trials are disrupted and future funding grants for non‐COVID‐19 projects have been reassigned or opened up to COVID‐19 only projects

TERM filed applies engineering and life science principles to develop methods to regrow, repair, or replace the damaged or diseased cells, tissues, or organs.^1^, ^2^, ^3^ TERM is a relatively new field and is just starting to be the most fascinating approach to develop new therapeutics at the dawn of the 21st century. Over the past few months, many scientists have been asked to stop their research. Many researchers have reported delays and disruptions to their clinical research. Hospitals have temporarily canceled their non‐urgent operations and clinical trials, to focus their precious resources on COVID‐19.

The tissue replacement and reconstructive surgeries are among the most canceled operation, including knee, hip replacement, as well as shoulder, ligament, and breast reconstruction. Indeed, the biggest recent advances in reconstructive surgeries over the past years are the result of TERM techniques, as these strategies have the potential to augment conventional treatment options in reconstructive surgeries. Just a few months after the COVID‐19 pandemic, clinical research staff running research in the areas of TERM are being made unavailable, and the recruitment of new participants to non‐COVID‐19 clinical trials are suspended, or significantly diminished. Meanwhile, many of the pharmaceutical and biotech companies have shifted their focus on the development of drugs and vaccines to treat people infected with this highly infective virus. It is expected that the COVID‐19 pandemic will have strong and yet possibly unexpected consequences and impact on the future funding of TERM research activities. TERM is an emerging field that developed over time and secure long‐term investment from both public and private sources is needed to help unlock the potential of TERM strategies and to boost research translation and commercialization in this area.

### 2.2 Virus infection leading to a remarkable reduction in tissue donation and extensive consideration for tissue storage

By having too many patients in the hospitals over the COVID‐19 outbreak period, the administration and process of cell and tissue donation programs have consequently slowed down. Moreover, concerns over the virus spread have led to a much smaller number of uninfected potential donors to be interested in donating their cells for research and therapies,^4^, ^5^ which further limit the already inadequate donor pool. Besides, warnings about social distancing, to stop the spread of a new coronavirus, are resulting in a significant drop in the tissue supply, specifically blood donation. In March 2020, the American Red Cross declared a severe blood shortage in response to the coronavirus outbreak. Also, during the COVID‐19 pandemic, many blood donation centers are closed around the world.^6^ To date, there are no reports on the SARS‐CoV‐2 transmission through the allogeneic blood transfusion, and future studies will clarify the risks of the transfusion‐transmitted virus.^6^

Also, the cells and tissues that have been donated and stored in tissue banks since the beginning of the COVID‐19 outbreak may already have been infected with this virus and have to be checked for the presence of 2019‐nCoV. Therefore, COVID‐19 testing has to be added to the already extensive list of infectious agents. Yet, 2019‐nCoV robust screening does not exist and there is an urgent need to develop a reliable test to screen samples upon harvesting for this highly infectious virus. Conduction of COVID‐19 tests enables us to ensure that cells and tissues are not contaminated by infectious agents before use.^5^

## 3 REGENERATIVE MEDICINE TECHNIQUES PROMISE TO ADVANCE HEALTH OUTCOMES IN COVID‐19 PATIENTS

As of 12 June, 2020, over 2100 clinical trials are officially registered for COVID‐19 treatment (ClinicalTrials.gov). These clinical trials range from the application of previously used antiviral drugs to novel therapies like cell therapies. At the same time, 169 “cell therapy” trials have been posted on ClinicalTrials.gov and a variety of cell sources, including mesenchymal stem cells (MSCs), are being used in trials to treat COVID‐19 patients. In most of these trials (156 studies), MSC therapy is used as a potential treatment for COVID‐19 (ClinicalTrials.gov).

### 3.1 Current status of MSC‐based therapies for acute respiratory distress syndrome

The protective effects of MSCs in the treatment of influenza respiratory infections have been reported previously in preclinical mouse studies.^7^, ^8^, ^9^ Intravenous administration of 5 × 10^5^ human bone marrow (BM)‐MSCs in aged H5N1‐infected immunocompetent mice reduced the viral‐associated acute lung injury and increased the survival rate.^8^ Li et al evaluated the effect of systemically administrated mouse BM‐MSCs (1 × 10^5^ cells) in the treatment of H9N2 AIV‐induced infection.^9^ The administration of MSCs reduced the cytokine storm and contributed to reduced H9N2 AIV‐induced acute lung injury and improved survival rate.^9^ Recently, Chen et al investigated the impact of intravenous implantation of allogeneic menstrual‐blood‐derived MSCs (1 × 10^6^ MSCs/kg body weight, three to four times injections) in 61 patients infected with the H7N9 virus in an open‐label clinical trial^10^ (Table 1). The intravenous infusion of MSCs lowered the mortality rate (17%) of H7N9 infected patients compared with the control group (54%).^10^ Since H7N9 and COVID‐19 both display similar complications (eg, acute respiratory distress syndrome [ARDS] and lung failure) and corresponding multiorgan dysfunction, therefore, MSCs therapy could be applied to COVID‐19. Liang et al treated a critically ill 65‐year‐old female COVID‐19 patient with umbilical cord‐derived MSCs (intravenous infusion, three times [5 × 10^7^ cells each time])^12^ (Table 1). In this case study, no obvious adverse effects were noted, and the patient was transferred out of the ICU 2 days after the third infusion. However, this case report was limited to only one patient, further investigations are required to support the positive outcome.

**TABLE 1:** List of clinical application of mesenchymal stem cells and their derivatives in patients with acute respiratory distress syndrome (ARDS)

Reference	Study phase and disease	Therapy	ARDS level	Route of administration	Country
Chang et al^11^	Case study (59‐year‐old man with pneumonia)	UCB‐MSCs	NR	Intratracheal administration	South Korea
Liang et al^12^	Case study (65‐year‐old female with COVID‐19)	UC‐MSCs	Sever	Intravenous infusion three times (5 × 10^7^ cells each time)	China
Simonson et al^13^	Case study (two patients; 58‐year‐old man, and 40‐year‐old man, with infection)	Allogenic BM‐MSCs	Sever	Intravenous administration (2 × 10^6^ cells/kg of body weight)	Sweden
Sengupta et al^14^	Prospective nonrandomized open‐label trial on the treatment of 24 COVID‐19 patients	Exosomes (ExoFlo) derived from allogeneic BM‐MSCs	Moderate‐severe	15 mL intravenous dose of ExoFlo	United States
Zheng et al,^15^ NCT01902082	Phase I; a randomized, placebo‐controlled pilot study. Twelve adult patients	Allogeneic AD‐MSCs	NR	Intravenous administration (1 × 10^6^ cells/kg of body weight)	China
Wilson et al,^16^ NCT01775774	Multicenter, open‐label, dose‐escalation, phase I clinical trial. Nine patients	Allogeneic BM‐MSCs	Moderate to severe	Three patients with low‐dose MSCs (1 × 10^6^ cells/kg of body weight), three patients received intermediate dose MSCs (5 × 10^6^ cells/kg of body weight), three patients received high dose MSCs (1 × 10^7^ cells/kg of body weight)	United States
Leng et al^17^	Phase I, in seven patients with COVID‐19 pneumonia	ACE2‐MSC		Intravenous administration (1 × 10^6^ cells/kg of body weight)	China
Yip et al,^18^ ISRCTN52319075	Phase I, in nine patients. Pneumonia and others	Wharton's jelly‐MSC	Moderate‐severe	Intravenous administration. Three patients received low‐dose MSCs (1.0 × 10^6^ cells/kg), three patients with intermediate dose (5.0 × 10^6^ cells/kg), and three patients with high dose (1.0 × 10^7^ cells/kg)	Taiwan
Bellingan et al,^19^ NCT02611609	Two open‐label, dose‐escalation cohort (n = 3 each). A subsequent, double‐blinded randomized subjects (n = 20) or placebo (n = 10)	Allogeneic BM‐MSCs	Moderate‐severe	Intravenous administration, 300 and 900 million cells.	United Kingdom/United States
Chen et al,^10^ ChiCTR‐OCC‐15006355 and NTC02095444	Phase I/II non‐randomized. Forty‐four patients with H7N9‐induced ARDS	Allogeneic Menstrual blood‐MSC from a healthy female donor (age 20‐45)	Moderate‐severe	Multiple intravenous infusion. Some patients were treated with three infusions of MSCs, and some received four infusions of MSCs. One million per kilogram of body weight for each time.	China
Liu et al,^20^ NCT01775774 and NCT02097641	A multicenter Phase I/II. Phase I: three cohorts of three patients	BM‐MSCs	Moderate‐severe	Single intravenous infusion. Phase I: Patients were received either 1 × 10^6^ cells/kg body weight (first cohort), 5 × 10^6^ cells/kg body weight (second cohort), or 10 × 10^6^ cells/kg body weight (third cohort). Phase 2: a randomized, double‐blind placebo (Plasma‐lyte A)‐controlled study using up to 10 × 10^6^ cell/kg body weight.	United States
Matthay et al,^21^ NCT02097641	Phase IIa. Prospective, double‐blind, multicenter, randomized trial. Sepsis, pneumonia, Aspiration. Forty patients received MSCs and 20 patients received placebo	Allogeneic BM‐MSC from three donors (aged 18‐45 years, one woman and two men)	Moderate‐severe	One dose of intravenous infusion. 10 × 10^6^/kg predicted bodyweight MSCs or placebo (Plasma‐Lyte A).	United States

Abbreviations: AD, adipose tissue‐derived; BM, bone marrow; MSCs, mesenchymal stem/stromal cells; NR, Not reported; UC, Umbilical cord; UCB, umbilical cord blood.

Wilson et al conducted a multicenter, open‐label, dose‐escalation, phase 1 clinical trial investigating the safety of a single dose intravenous injection of allogeneic BM‐MSCs in patients with moderate to severe ARDS (n = 9) (ClinicalTrials.gov: NCT01775774).^16^ Patients were treated with low‐dose MSCs (1 × 10^6^ cells/kg predicted body weight [PBW], n = 3), or intermediate dose MSCs (5 × 10^6^ cells/kg PBW, n = 3), or high dose MSCs (1 × 10^7^ cells/kg PBW, n = 3). Results showed that a single infusion of up to 10 million cells/kg PBW was well tolerated and no infusion‐associated events or MSCs treatment‐related adverse events were reported.^16^ In the weeks after infusion, serious adverse events were noticed in three patients. One patient died on day 9, one patient died on day 31, and one patient was discovered to have multiple embolic infarcts of the brain, kidneys, and spleen, but thought to have occurred before the MSC administration based on MRI results. The authors concluded these severe adverse events were not related to MSCs treatment.^16^ In a double‐blind, multicenter, randomized phase 2a safety trial, 40 patients with moderate to severe ARDS were treated with BM‐MSCs (10 × 10^6^/kg PBW) and compared with the placebo group (n = 20) (NCT02097641).^21^ The patients were treated within 7 days of ARDS diagnosis. The 28‐day mortality rate was not significantly different between the MSC‐treated group (30%) and the placebo group (15%). The BM‐MSCs treated group had numerically higher mean scores for Acute Physiology and Chronic Health Evaluation III than the placebo group.^21^ Still, the sample size in this trial was too small to reliably assess the efficacy of MSCs therapy in ARDS and larger trials are needed.

**FIGURE 1:**
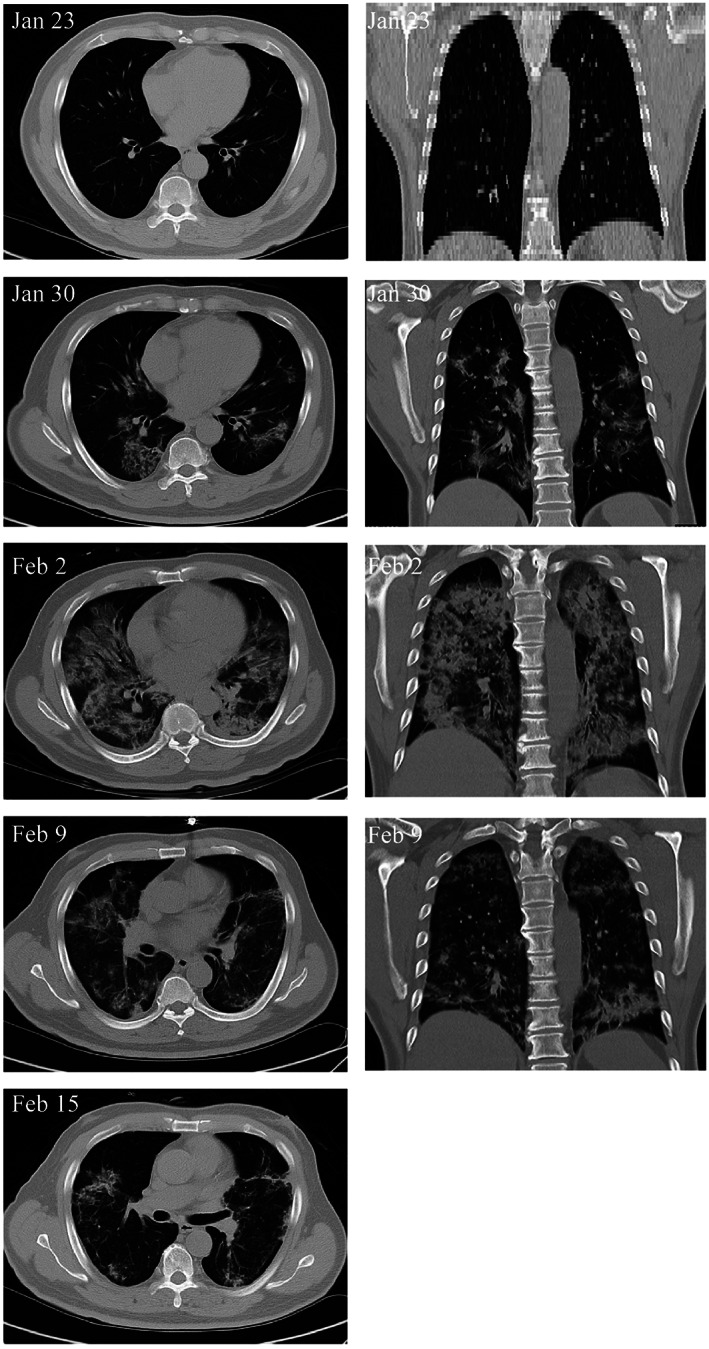
Transplantation of mesenchymal stem cells (MSCs) improves the outcome of patients with COVID‐19 pneumonia. Chest computerized tomography imaging showed that the COVID‐19 pneumonia was largely reduced on day 9 after MSC transplantation, adapted with permission from Leng et al^17^

A recent study was conducted to investigate the impact of “angiotensin I converting enzyme 2” receptor‐negative (ACE2^−^) MSCs for the treatment of COVID‐19 patients (Figure 1).^17^ ACE2 has a central role in the pathogenesis of COVID‐19 and is expressed in the surface of human cells, especially the alveolar type II cells and capillary endothelium.^22^ The intravenous infusion of MSCs (1 × 10^6^ cells/kg weight) resulted in an increase in peripheral lymphocytes, a decrease in the C‐reactive protein, and overactivation of CXCR3+CD4+ T cells, and CXCR3+CD8+ T cells. Moreover, MSC therapy showed a significant reduction in the TNF‐α levels and an increase in IL‐10 levels compared to the placebo control group. No toxicities, allergic reactions, secondary infections, or severe attributable adverse events after MSC administration were observed.^17^ MSC administration resulted in improved functional outcomes in the patient with critically severe conditions (n = 1). Nevertheless, Leng et al study^17^ was limited as these case series were not randomized nor blinded, and while detailed information for only one critically ill patient (out of seven) was provided, no corresponding information was given for the other six patients (severe [n = 4] and common type [n = 2]) or for the placebo patients (n = 3). To determine the potential benefits of MSCs for COVID‐19 and understand the mechanisms of action of MSCs, more detailed information including the timing of MSC administration relative to the onset of disease, and cell populations for both MSCs treated and placebo patients are required.^7^

In COVID‐19 infection, the host immune system produces an enormous inflammatory response in an attempt to kill the virus, leading to a severe cytokine storm, this process is the main contributor to organ damage in COVID‐19. Therefore, avoiding the cytokine storm could be an effective strategy in the treatment of COVID‐19. MSCs, due to their powerful immunomodulatory ability not only suppress the cytokine storm but also promote the endogenous repair/regenerative mechanisms in the lungs after the COVID‐19 infection.^17^, ^23^

ARDS is one of the most severe complications caused by coronaviruses.^24^, ^25^ Indeed, respiratory failure from ARDS is the leading cause of mortality in COVID‐19 patients.^24^, ^25^ Therefore, the management and treatment of ARDS are essential to reduce the mortality rate. It is believed that MSCs regulate the immune system by inhibiting the production of inflammatory cytokines by lymphocytes and induces the production of anti‐inflammatory cytokines. Therefore, MSC therapy potentially offers a unique therapeutic option to help COVID‐19 patients. However, a greater body of research is required to fully evaluate the potential of MSCs therapy for COVID‐19 induced ARDS.

### 3.2 The potential use of stem cell derivatives for ARDS treatment

Apart from MSCs, their derivatives including exosomes have gained significant attention as a therapeutic strategy to achieve the therapeutic effects of MSCs without the risks and challenges of administering the cells to the patients.^26^, ^27^ Exosome‐based therapy is an emerging field of biomedical research and the therapeutic effects of stem cells have been attributed to the secreted exosomes.^28^, ^29^ Specifically, the potential use of exosomes to ameliorate ARDS in preclinical models is well‐documented.^27^ To date (June 13, 2020), 174 clinical trials have been registered to utilize exosomes within a range of therapeutic applications (Clinicaltrials.gov). However, there are no FDA‐approved exosome products available on the market yet and each product requires an individual IND for each trial.

Previous studies mainly administered exosomes by direct injection into the injured tissues or by intravenous injection, but inhalation could be the most direct and less invasive route of delivery to treat COVID‐19 induced ARDS. Recently, Dinh et al reported the lung spheroid cell‐secretome (soluble proteins, and extracellular vesicles) and exosomes delivered by inhalation could resolve the bleomycin‐ and silica‐induced lung fibrosis by reestablishing the normal alveolar structure.^28^ A new registered trial (NCT04276987) aims to investigate the safety and efficiency of aerosol inhalation of allogeneic adipose tissue‐derived MSCs' exosomes in the treatment of severe COVID‐19 patients. Another trial aims to test the safety and efficacy of exosomes derived from allogenic T cells following targeted delivery by metered‐dose inhaler in 60 participants (NCT04389385). Future clinical trials using exosomes and the conditioned media produced from MSCs might be an effective therapeutic for coronavirus‐induced ARDS.^30^

### 3.3 The alternative sources of stem cells

In the field of cell therapy for ADRS, a challenge is to identify appropriate cell populations that are able to modulate alveolar stem cell function and regulate the hyperinflammation caused by a cytokine storm. The use of human tissues like placental tissues is less ethically controversial and allows the isolation of relevant quantities of stem cells for mainstream clinical applications.^31^ Since the onset of the COVID‐19 outbreak, the application of MSCs from different sources is proposed, including but not limited to menstrual blood MSCs (ChiCTR2000029606), umbilical cord MSCs (NCT04269525), cord blood MSCs (ChiCTR2000029816), and dental pulp MSCs (NCT04302519) (CellTrials.org). For some trials, no information was provided regarding tissue source or mode of manufacturing. In the application of MSCs for infectious diseases, the source of MSCs, dose, administration route, and dosing strategies (including the number and timing of administrations) should be meticulously investigated.^7^

The field of regenerative medicine (RM) has experienced uncertainty in regulatory review since the beginning. RM products are different from other clinical products, while RM products provide benefits in terms of healing and regeneration but the long‐term effects in human hosts are unknown. Therefore, introducing a new RM product to the market involves numerous phases of clinical testing, which can require more than a decade of development and testing.^1^ Although hopes for MSC therapies in COVID‐19 patients are high, there is currently little available data on whether human cells are safe and effective for treating COVID‐19. The limited access to autologous products and inadequate diverse donor pools are other major barriers in the application of RM therapies for COVID‐19.

## 4 APPLICATION OF 3D TISSUE MODELS TO UNDERSTAND VIRUS‐CELL INTERACTIONS AND TEST DRUGS AGAINST HIGHLY INFECTIOUS VIRUSES, INCLUDING COVID‐19

**FIGURE 2:**
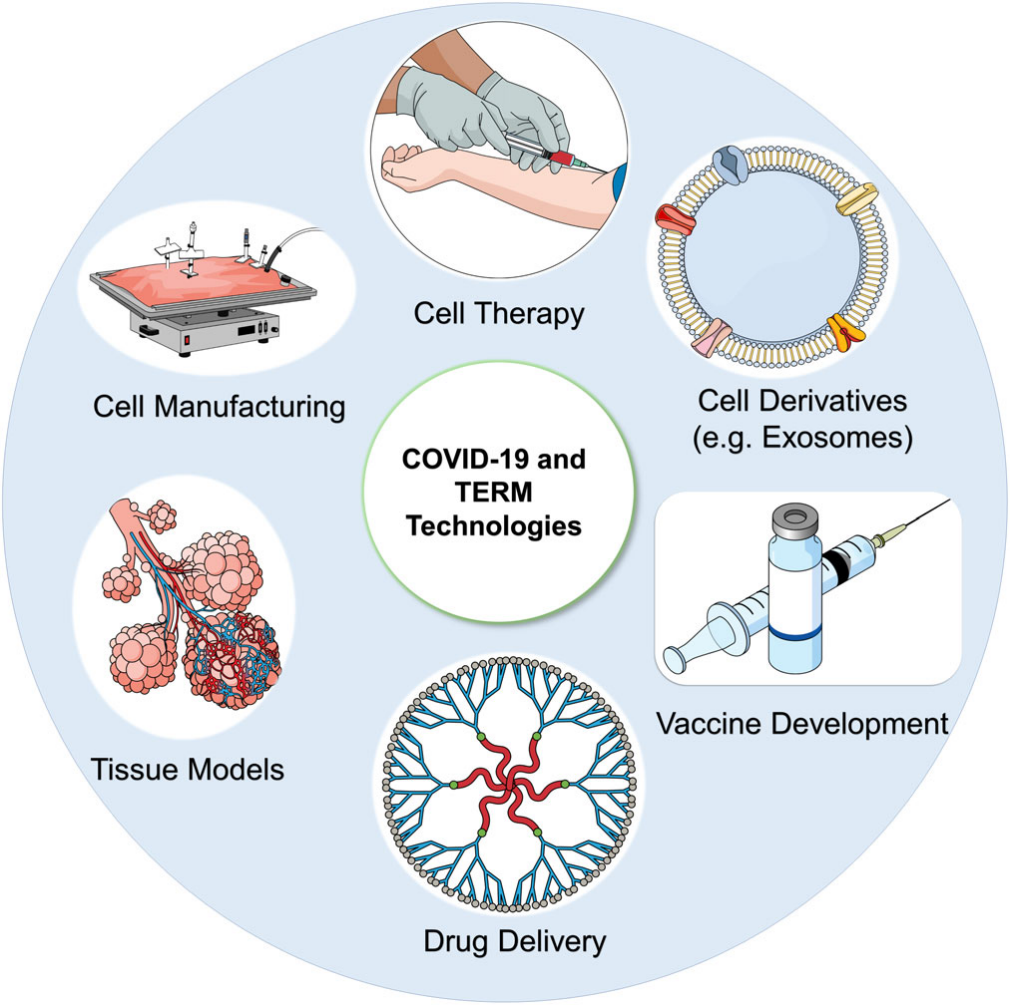
Coronavirus disease 2019 (COVID‐19): a tissue engineering and regenerative medicine perspective (TERM). The TERM concepts have been used to develop three‐dimensional platforms to understand virus‐cell interactions and test drugs against COVID‐19. Besides, the biomaterials could be used to develop vaccines or as drug delivery systems

During COVID‐19 pandemic, development of robust and reliable tissue‐engineered 3D models as platforms for the screening of antiviral therapeutics, precise understanding of the mechanism of COVID‐19 disease, and the host and SARS‐CoV‐2 interactions would be of value (Figure 2). The potential of 3D engineered constructs as robust tools for the assessments of viral pathogenicity and identifying host responses to antiviral therapy has been tested in previous studies.^32^, ^33^

### 4.1 In vitro infection models

Indeed, conventional cultures cannot form specific tissue structures as they exist in the living human organs. Early 3D attempts utilized explant tissue cultures of the human respiratory tract to circumvent the limitations associated with 2D cell culture.^34^ However, explant cultures are limited due to the donor availability and their infeasibility in long‐term cultures.^35^ Suderman et al designed a 55‐mL rotating wall vessel (RWV) bioreactor to generate human bronchial‐tracheal mesenchymal cells/bronchial epithelial (BEAS‐2B) cells‐derived 3D tissue‐like assembly (TLA) (aggregates, 2‐3 mm in size) to assess the pathogenicity and infectivity of SARS‐CoV‐1.^36^ They used Cultispher G beads as the main support matrix for cell attachment in RWV. Histological and morphological studies at day 10 postincubation of 3D‐LTA aggregate with the SARS‐CoV showed swollen mitochondria, a significant decrease in the number of mitochondria, an increase in the number of cytoplasmic vacuoles, the disruption of endoplasmic reticula networks and eventually shedding off the cells in microcarrier beads that are the pathological signs of the 3D‐TLA aggregates infection by SARS‐CoV.^36^

A recent study aimed to assess SARS‐CoV‐2 infectivity.^22^ Spike (S) protein of SARS‐CoV‐2 is composed of two subunits S1 and S2. S1 binds to ACE2 protein, the key entry gate for SARS‐CoV‐2 that facilitates its penetration into target cells, and S2 fuses on the surface of the cell membrane.^37^ Transmembrane serine protease 2 (TMPRSS2) is another host protein that promotes cellular entry of SARS‐CoV‐2. Therefore, both ACE‐2 and TMPRSS2 are necessary for viral infectivity.^22^ ACE2 is highly expressed in pneumocytes types I and II and renal proximal tubule epithelial cells as well as vascular endothelial cells and small intestinal epithelial cells. The SARS‐CoV‐2 acts in a highly selective manner to spread and replicate, which is human cell‐specific; therefore, the application of cells and proteins is critical.

Monteil et al demonstrated that SARS‐CoV‐2 could directly infect capillary organoids and kidney organoids generated from human induced pluripotent stem cells.^38^ They showed the use of clinical‐grade human recombinant soluble ACE2 (hrsACE2) specifically stopped viral penetration and prevented viral infection.^38^ The current gold standard method for screening anti‐SARS‐CoV‐2 therapeutics is using human stem cells‐derived capillary and kidney organoids and monolayer Vero E6 cell culture. Monolayer culture of Vero E6 cells with collected supernatant of infected organoids showed rapid SARS‐CoV‐2 replication.^38^ in vitro treatment with hrsACE2 substantially decreased the SARS‐CoV‐2 infection in the engineered human organoids in a dose‐dependent manner. These results indicate the hrsACE2 can inhibit SARS‐CoV‐2 infection and could be considered as a potential therapy for COVID‐19.^38^ Another report established human intestinal organoids using multipotent adult tissue MSCs and showed robust SARS‐CoV‐2 replication in human intestinal organoids.^39^ The potential use of human and animal organoids as an experimental virology platform has been discussed somewhere else.^40^, ^41^

Human organ‐on‐chip technology has been developed and extensively used to recapitulate in vivo cellular responses to drugs or toxic agents.^32^, ^42^, ^43^ The tissue engineering and organ‐on‐chip technologies apply engineering principles to biological processes and enable rapid translation of technologies from the benchtop to the bedside.^44^ Previous studies reported lung‐on‐chip models to offer alternative preclinical tools to mimic human alveolar epithelial cells' responses to viral infection due to their capacity to recapitulate organ‐level physiology and pathophysiology.^45^, ^46^ Si et al fabricated a microfluidic device with two microchannels separated by a porous membrane.^32^ Basal stem cells and respiratory epithelial cells were cultured on both sides of the membrane and then exposed to the synthetic pseudovirions composed of SARS‐CoV‐2 S protein. Increasing the number of virus‐infected epithelial cells indicating the development of basal stem cells into the pseudostratified mucociliary epithelium, and SARS‐CoV‐2 penetration to lung epithelial cells through binding to ACE2 receptor expressed on the epithelial cell surface in microchannels. Then, multiple FDA‐approved SARS‐CoV‐2 inhibitors, including chloroquine, arbidol, toremifene, clomiphene, amodiaquine, verapamil, and amiodarone, were perfused through the channels. Results showed only amodiaquine and toremifene were able to prevent SARS‐CoV‐2 infection.^32^ Of significance, no significant inhibitory effects for chloroquine was observed in Cmax. This could be explained by the fact that chloroquine may exert its therapeutic effects via mechanism(s) other than contributing to blocking the virus entry.^32^

Remdesivir (also known as GS‐5734) is an adenosine analog prodrug with excellent potency against severe acute respiratory syndrome and the Middle East respiratory syndrome in human airway epithelial (HAE) cell models.^34^, ^47^ Previous studies showed remdesivir might be a promising candidate for the treatment of patients with COVID‐19 due to its ability to inhibit viral RNA replication.^48^ HAE cultures are the most preclinically relevant models for lung tissue that simulate the physiological and morphological features of the respiratory epithelium in the human conducting airway. In this regard, Agostini et al cultured human alveolar cells on the permeable Transwell‐collagen membrane, in an air‐liquid interface, to mimic in vivo respiratory epithelium and evaluated the therapeutic effects of the nucleotide prodrug GS‐5734 against SARS‐CoV infection.^47^ The addition of GS‐5734 resulted in decreased SARS‐CoV titer during 48 and 72 hours posttreatment without measurable cellular toxicity in HAE cultures. The results support the utility of this tissue model for studying the inhibitory effect of GS‐5734 against SARS‐CoV.^47^

In another study, human octamer‐binding transcription factor 4+ (Oct‐4+) progenitor cells and MSCs were grown on a collagen type I‐based matrix in a serum‐free media.^49^ The authors reported progenitor cells were able to differentiate into alveolar pneumocytes type I and II expressing ACE2. The results displayed that the Oct‐4+ progenitor cells, but not the surrounding mesenchymal cells, were susceptible targets for SARS‐CoV‐1 and allowed virus replication.^49^ This suggests an important potential role for Oct‐4+ progenitor cells, which normally develop into the cilia in the bronchi and are mainly responsible for expressing ACE2, in the continued destruction of alveoli and loss of the lung capacity for regeneration after SARS‐CoV infection.^49^ Despite significant advances, a major drawback of current 3D lung models is the lack of human stromal, hematopoietic, and immune systems, as the critical components in the viral infection and pathogenesis. Therefore, future 3D models should investigate the safety and efficacy of antiviral agents in the presence of human immune and hematopoietic systems.

## 5 BIOMATERIAL‐BASED VACCINES

Apart from the development of in vitro models, tissue engineering technologies enable the evolution of the next generation of drug delivery systems and facilitate vaccine development and delivery.^43^, ^44^ Tissue‐engineered systems allow the controlled extended release of drugs, which are advantageous over multiple injections for clinical practice. Besides, the new generation of biomaterials allows us to target the areas of high viral load specifically and extendedly. Biomaterials able to act as the drug delivery vehicle for the vaccine as well as the adjuvant, and can boost the immune response to the vaccine.^44^, ^50^ For H5N1 influenza immunization, Wu et al modified chitosan and developed a thermal‐sensitive hydrogel as an intranasal vaccine delivery system.^51^ The new adjuvant‐free vaccine delivery system prolonged the H5N1 split antigen residence time in the nasal cavity and enhanced the transepithelial transport in the nasal epithelial tissue. The adjuvant‐free vaccine delivery system could induce larger antigen‐specific systemic immune responses and mucosal IgA immunity in a mouse model.^51^ In addition, the tissue engineering concepts have been utilized to develop immunologically active biomaterial constructs.^52^ Ali et al fabricated 3‐dimensional, macroporous poly(lactide‐co‐glycolide) matrices that slowly released cytokines such as granulocyte‐colony stimulating factor and recruited antigen‐presenting cells to the matrices.^52^ The biomaterial‐based vaccines enhanced effective, prolonged, and specific cytotoxic, T‐cell mediated immunity, and eradicated the large established melanoma tumors in mice.^53^ The in vivo modulation of host immune cells can be achieved with the spatiotemporal control of biochemical and mechanical cues in biomaterials.^54^ Mesoporous silica rods of high aspect ratio were fabricated and subcutaneously implanted into mice to form a pocket and formed 3D interparticle spaces and recruited host cells. The sustained release of inflammatory signals and adjuvants from the scaffold modulated the immune cell function and provoked adaptive immune responses.^54^ This system has been applied to tumor vaccines in animal models with promising results,^52^, ^55^ and may serve as a platform for the design and development of vaccines against SARS‐CoV‐2.

## 6 CHALLENGES AND OPPORTUNITIES IN CLINICAL MANUFACTURING OF REGENERATIVE MEDICINE PRODUCTS FOR COVID‐19

### 6.1 Cell manufacturing requirements during pandemic

The current industry standard for the manufacturing of adherent therapeutic cells, such as MSCs, relies on planar technologies, that is, flask systems. For MSCs therapeutic products in clinical development, typical planar systems used can range from T225 to multilayer vessels. Ten‐layer vessels such as the Corning CellStack or ThermoFisher Nunc Cell Factory are common platforms adopted for larger production typically required in phase II/III trials. However, for the cultivation of adherent adult cells, planar systems are capped at 15 billion to 20 billion cell lot sizes unless significant automation is applied.^56^ In the case of MSCs, typical cell densities at 80% confluence in serum‐based media is around 25 000 cells/cm^2^ ^56^ and up to 35 000 cells/cm^2.^ ^57^ In a 10‐layer vessel, which has a surface area of around 6400 cm^2^, the average cell number obtained from a single culture vessel would be around 160 million to 224 million (M) cells. However, planar technologies will need to scale‐out, and this starts to become very challenging when running manufacturing lot sizes beyond 50 to 70 vessels each time. Robotics and automation technologies can be implemented but they are often very expensive, and not available at most good manufacturing practices (GMP) facilities. In a pandemic like COVID‐19, the ability to rapidly deploy large quantities of MSCs to treat patients with severe symptoms of COVID‐19 is paramount. What capabilities do we have today to meet such demand?

### 6.2 Are planar technologies scalable?

To estimate the required manufacturing lot sizes for treating the patients in the pandemic, it is necessary to factor in cell loss during downstream processing, postthaw recovery, and those required for QC testing. By a reasonable estimation, the manufacturing requirements to deploy human MSC (hMSC) doses for 10 000 patients at a target cell dose of 300 M cells per patient is around 6.4 Trillion cells (9). Using 10‐layer vessels that can produce around 10 billion cells per manufacturing lot (using 40 vessels), it would require around 640 lots to achieve this target number. Realistically, even with the aid of automation, this manufacturing operation would take a very long time (years) to complete.

Alternate technologies for culturing adherent cells include packed‐bed bioreactors, which may consist of beads, porous structures, or hollow fibers. One such system that supports the manufacture of clinical‐grade hMSCs is the Quantum Cell Expansion System, which provides a total surface area of 2.1 m^2^ per bioreactor. Typical harvests achieved for BM‐MSCs in the Quantum bioreactor range between 220 M^58^ to 660 M^59^ and up to 1B cells^60^ over 7 to 21 days of expansion. In the context of manufacturing for COVID‐19, a single bioreactor run using the Quantum Cell Expansion System is only sufficient to make a single dose to treat one patient. Cell production at larger scales using such devices will require parallel systems to be in operation.

To achieve lot sizes in the hundreds of billions to trillions of cells, it would require manufacturing platforms that can scale‐up not just scale‐out. Suspension bioreactors utilizing microcarriers to support the growth of MSCs is an attractive option. Microcarrier‐based bioreactor cultures can benefit in media productivity over traditional flask systems due to the large surface area to volume ratio and enable precise control plus monitoring capabilities of key process parameters like gas concentration, nutrient levels, and pH measurements.^56^ To this end, various groups have characterized and optimized culture parameters for efficient expansion of hMSCs in suspension bioreactors. Typical expansions achieved in spinner flasks can range from 90 to 170 M cells/L. One group reported achieving 270 M cells in a 3L single‐use spinner‐flask bioreactor over 5 days.^61^ Rafiq et al reported that they can achieve 850 M in a 5‐L stirred‐tank bioreactor over 12 days.^62^ An alternative to traditional spinner flasks is suspension bioreactors like the PBS vertical‐wheel bioreactor. Kalra et al reported the expansion of adipose‐derived MSCs in PBS mini 0.5 L bioreactors in the range of 76 M to 100 M cells. Based on current observations, microcarrier‐based cultivation of hMSCs in suspension bioreactors could generate 50 to 200 000 cells/mL. Paired with a highly robust hMSCs stock and high‐performance expansion media systems, Kirian et al have reported achieving cell densities of greater than 0.5 M cells/mL in 4 to 5 days at various development scales in the PBS Biotech bioreactors: from small scale (0.5L) to development (3L), to pilot (15L) and production (50L) scale.^63^ At the 50L scale, this generated lot sizes ranging from 25 billion to 30 billion in a single run, and if scaled to 500 L then this would yield lot sizes north of 250 billion cells—a true commercially relevant lot size.

While microcarrier‐based cultivation allows us to scale‐up, another challenge to keep in mind for clinical manufacturing is the downstream processing steps. The important considerations here, as described by Olsen et al are the cell harvesting steps, cell separation steps, and fill‐finish steps.^64^ Particularly when looking at concentrating many liters of cell solution, this must be done promptly (<5 hours) to not compromise cell viability and the functionality of the final product.^64^ Volume reduction and filtration steps using appropriate systems at the scale that is required will need to be evaluated accordingly. Most standard laboratory centrifugation systems will not be able to handle large volumes >5 to 10 L.^56^ In the separation step, the choice of the enzyme, the time of exposure, and efficiency of each step are critical in ensuring optimal cell survival. For GMP manufacturing, the use of animal‐component free materials, in a ready‐to‐use solution, is preferred and helps to reduce regulatory burden.

### 6.3 What can we expect to learn from this pandemic (with new clinical trials approved)?

Acceleration of trials for COVID‐19 will present opportunities to collect more data on both the safety and efficacy of MSCs and other cell therapies to treat lung injuries and related complications. However, rational design and a controlled approach to clinical trial design are essential to obtain valuable insights in assessing both the safety and efficacy of treatments.^65^ Patient safety is paramount and should never be compromised in any circumstance. Therapeutic developers and cell therapy manufacturers must uphold their moral integrity to deliver products that meet equally stringent requirements to ensure patient safety. From the manufacturing perspective, this also presents an opportunity for product developers to focus on advancing scalable technologies that can meet critical demands like these in the future. As other cell therapy products are developed and mature, manufacturing will remain a challenge if progress is not made on this front. Finally, the cost to manufacture cell therapies will need to go down to make them accessible to everyone. In the Olsen et al article, the relationship between technology S‐curve and economics for MSCs describes how the adoption of each technology platform at the production scale will drive the cost down due to efficiencies of scale.^64^ The COVID‐19 pandemic is forcing us to think big and look ahead into the future. With every crisis, there is a silver lining, and here we are being presented the opportunity to advance cell manufacturing technologies forward to make cell therapies of the future scalable, safe, and affordable.

## 7 CONCLUSIONS

The TERM technologies have the potential to revolutionize the whole healthcare system by restoring damaged tissues and organs, in contrast to other pharmaceuticals and surgical strategies that generally manage rather than cure diseases.

Over the COVID‐19 outbreak, the funding for many TERM projects is being cut, which has a significant impact on the present and future of TERM research and therapies. Meantime, many research institutes and pharmaceutical companies in this area repurposed their technologies and developed new research programs to understand the interaction of viruses and human tissues or develop vaccines and therapies for COVID‐19. ARDS is the principal cause of death in COVID‐19 infection and MSCs therapies have been applied to treat the COVID‐19 patients. One of the main challenges in the administration of MSCs for COVID‐19 is the limited number of autologous MSCs sources and limited timelines to treat accelerating case numbers. Therefore, application of a safe off‐the‐shelf, allogeneic product, with appropriate immune response modulation activity, would allow patients to receive the best quality stem cell treatments at the right time and effective clinical densities.

Current clinical trials highlight the potential benefits of stem cell therapies for COVID‐19 patients. However, current studies are made up of small case series that lack appropriate control arms and historical controls were not provided, making the interpretation of any reported benefits difficult to quantify. Therefore, further investigations are required to understand the safety and efficacy of these therapies and their long‐term outcomes. Effective multi‐institutional collaboration and adequate funding from government and non‐government sources are also needed to collect and analyze the data from ongoing and new human trials, to better understand the potential benefits of stem cell therapies for COVID‐19 patients.

## CONFLICT OF INTEREST

M.L. declared employment with RoosterBio. The other authors declared no potential conflicts of interest.

## AUTHOR CONTRIBUTIONS

A.S.: concept and design, manuscript preparation, collection and assembly of data, review and editing, and final approval of the manuscript; L.M., M.L., J.B.: manuscript preparation, discussion, and review and editing.

## Data Availability

Data sharing is not applicable to this article as no new data were created or analyzed in this study.
